# Embedding lived experience into mental health academic research organizations: Critical reflections

**DOI:** 10.1111/hex.13586

**Published:** 2022-08-23

**Authors:** Lisa D. Hawke, Natasha Y. Sheikhan, Nev Jones, Mike Slade, Sophie Soklaridis, Samantha Wells, David Castle

**Affiliations:** ^1^ Centre for Complex Interventions Centre for Addiction and Mental Health Toronto Ontario Canada; ^2^ Department of Psychiatry University of Toronto Toronto Ontario Canada; ^3^ Dalla Lana School of Public Health University of Toronto Toronto Ontario Canada; ^4^ School of Social Work University of Pittsburgh Pittsburgh Pennsylvania USA; ^5^ School of Health Sciences University of Nottingham Nottingham UK; ^6^ Faculty of Medicine & Health Sciences Nord University Namsos Norway; ^7^ Education Services Centre for Addiction and Mental Health Toronto Ontario Canada; ^8^ Institute for Mental Health Policy Research Centre for Addiction and Mental Health Toronto Ontario Canada

**Keywords:** academics, addiction, lived experience, lived expertise, mental illness, patient engagement, patient‐oriented research

## Abstract

**Background:**

As part of a growing emphasis on engaging people with lived experience of mental health conditions in mental health research, there are increasing calls to consider and embed lived experience throughout academic research institutes. This extends beyond the engagement of lay patients and also considers the potential roles of academic researchers with lived experience. When the lived experience of academic researchers is applied to academic work, there is the potential to improve the relevance of the research, while destigmatizing mental illness within academia. However, there are different and often contrasting perspectives on the way a lived experience academic researcher initiative should be implemented.

**Objectives:**

This article describes some of the key issues to be considered when planning an initiative that leverages and values the lived experience of academic researchers, including the advantages and disadvantages of each potential approach.

**Discussion & Recommendations:**

Institutions are encouraged to reflect on the ways that they might support and value lived experience among academic researchers. In developing any such initiative, institutions are encouraged to be transparent about their objectives and values, undertake a careful planning process, involve researchers with lived experience from the outset and consistently challenge the stigma experienced by academic researchers with lived experience.

**Patient or Public Contribution:**

Multiple authors are academic researchers with lived experience of mental health conditions.

Research communities have begun to place greater emphasis on involving people with lived experience of a health condition in research about that condition, often as full partners. This approach is known as patient‐oriented research (POR),^1^, ^2^ also described as patient and public involvement in research. It is proposed that research using a POR approach will have heightened relevance, feasibility, adoption, implementation and sustainability.^1^ POR is valuable across domains of health research,^3^ including mental health and substance use.^4^

Many highly productive academic researchers across disciplines live with chronic illnesses, including mental illnesses,^5^ and this is not a new phenomenon. Viewed dimensionally, mental illness includes common as well as rarer mental disorders and mental health conditions, with experiences ranging from mild to severe. While some people with mental illness live with severe disabilities that prevent certain typically recognized achievements, others have been successful in obtaining academic qualifications and research career success.^6^, ^7^ Since personal meaning often guides career decisions,^8^ it is natural that people with lived experience may be drawn to the helping sciences, including research; conversely, high demands within academia may lead to the emergence of symptoms. High rates of depression, anxiety, distress and suicide among populations like physicians and graduate students demonstrate that lived experience is among us, whether outwardly acknowledged or not.^9^, ^10^ Through personal disclosure publications, an increasing number of academic researchers have been acknowledging their lived experience and sharing their journeys.^6^, ^7^, ^11^, ^12^, ^13^, ^14^, ^15^

When lived experience is fully recognized within mental health research spheres, an opportunity is afforded to pair POR approaches with the recognition of lived expertise at all levels of research, including research leadership, to generate research guided by lived experience. Indeed, there have been calls for the explicit engagement of lived experience among academic researchers and examples of success,^6^, ^7^, ^13^, ^16^, ^17^, ^18^ including in dedicated lived experience roles and in traditional research roles to which researchers may bring their lived experience. However, lived experience integration in research comes with challenges, barriers and criticisms.^19^ Self‐relevant research, dubbed ‘me‐search’, has been criticized as being biased and nonobjective.^20^ This criticism is in direct conflict with POR philosophies.^1^ This conflict demonstrates the need for decision‐makers to reflect on how to embed and recognize lived experience among the body of academic researchers.

It is important to consider which mental illnesses are represented among lived experience academic researchers and who is missing. Some people with lived experience face disability‐related barriers and inequities that prevent them from achieving academic success,^17^ and are therefore underrepresented. Rates of mental illness and mental health conditions in academia are unclear in the context of limited disclosure and stigma; however, one survey found high rates of anxiety and depression, but very low rates of schizophrenia and substance use disorders, while failing to report on important diagnostic categories within the mental health sphere, such as personality disorders.^21^ Recognition that lived experience exists among academic researchers may have the power to dismantle stigma by demonstrating that lived experience and academic success are not mutually exclusive. At the same time, it is important to acknowledge that certain mental health conditions are underrepresented in academia,^21^ posing a challenge to equity, diversity and inclusion (EDI) efforts.

At the Centre for Addiction and Mental Health, we have explored recommendations regarding initiatives to integrate and value lived experience among academic researchers within a major research and teaching hospital. Based on the literature and informal stakeholder discussions, we present herein a nonexhaustive description of key considerations to contemplate before embarking on lived experience integration. These considerations are presented from our positionalities as mental health researchers at various career stages, some of whom have lived experience of mental health conditions. In this context, we recognize that there are many elements of valuable debate representing different experiences; we therefore provide multiple perspectives to stimulate discussion, not seeking to offer definitive solutions. Overarching concepts reflected herein are summarized in Figure 1.

**Figure 1:**
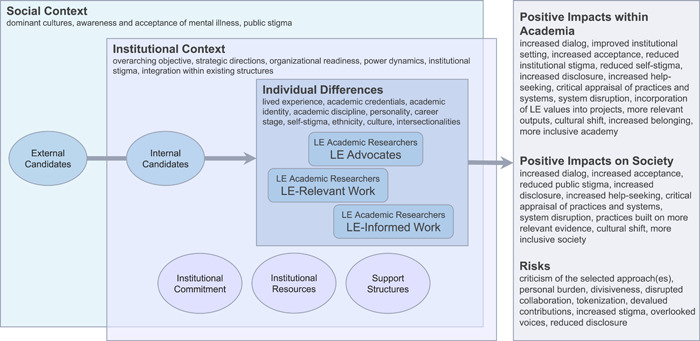
Factors affecting the embedding of academic researchers with lived experience into a research institution. LE, lived experience.

## 1 WHAT IS LIVED EXPERIENCE?

Lived experience is not a single, universally defined phenomenon. Among the wide range of experiences that individuals may have, each represents a personal life journey, with its own challenges, successes and personal meanings. Herein, when we refer to lived experience, we are inclusive of current and/or past substantial mental health challenges or conditions of considerable psychological distress, generally including one or more psychiatric diagnoses (inclusive of substance use disorders), often driven by multifactorial causality and accompanied by intersecting problems with stigma, discrimination, social determinants of health, impacts on functioning and quality of life and mental health service utilization.^17^, ^22^ Research institutions may acknowledge the full range of lived experience, from mild to severe, which is a thread throughout an organization, while also considering representation and EDI. Family members can also be included in definitions of lived experience, given the important role they can play in supporting a loved one. Defining lived experience as inclusive of family members would enable family members to represent loved ones whose lived experience has prevented them from achieving academic credentials, while also bringing learnings drawn from the process of supporting and caring for a loved one with lived experience.^23^, ^24^ While important, family member contributions should not supersede the voices of people who have personally and directly experienced mental health conditions.

## 2 WHAT IS A LIVED EXPERIENCE ACADEMIC RESEARCHER?

In our Western, urban context, an ‘academic researcher’ refers to a person who has attained a doctoral or other advanced degree, and who is working—or who aims to work—in an academic research role, generally within a university or hospital‐based academic setting. This can be inclusive of professors (any rank), scientists, clinician–scientists and postdoctoral research fellows. Extending this definition further to encompass earlier career stages, ‘academic researcher’ can sometimes include Ph.D. students, other doctoral candidates and individuals in analogous roles. Some may include in the definition people with master's degrees or master's students who are doing academically relevant work. While these are typical definitions of ‘academic researcher’, in the lived experience sphere, some might recommend attributing researcher titles to individuals with lived experience who do not have advanced degrees, adding ‘academic’ or ‘researcher’ to the range of other senior roles they may hold as industry leaders or activists.^25^

If an institution adheres to the definition requiring an advanced degree, lived experience academic researchers are dually qualified people who have both academic credentials and experiential expertise. On the one hand, maintaining the need for academic credentials might foster greater acceptance within the research community, as the existing academic body may recognize the lived experience researchers as having equivalent credentials, and thus as being ‘one of their own’. Institutional leaders may also be reassured that the integrity of academic structures has been maintained. However, this definition may be criticized by those who doubt the veracity of lived experience among researchers with traditional qualifications.^17^ A definition that does not require traditional academic qualifications would open the research space to new voices, while acknowledging that lived experience prevents some from entering the academy. Yet, this approach could also fuel stigma by reinforcing differences and denying the universality of lived experience.

## 3 WHY CONSIDER INTEGRATING LIVED EXPERIENCE WITHIN RESEARCH INSTITUTIONS?

While there may be many motivations for integrating lived experience into a research institution, through our consultations and experience, we have identified three overarching objectives: (1) to openly and critically appraise mental health systems and research to advocate for change; and/or (2) to improve research relevance and destigmatize lived experience within the field; and/or (3) to gain reputational benefits as a progressive institution that recognizes and values lived experience. Each of these objectives will depend on the institution's mission and contexts, such as strategic directions, organizational readiness, resources, support structures, power dynamics and stigma. They will also be influenced by the broader social context, dominant cultures, research funder preferences and the level of public awareness, acceptance and stigma of mental illness.

The first objective has the potential to disrupt systems in positive ways, forcing shifts within academia and beyond by critiquing the status quo and providing new perspectives. There is, however, a risk of negative disruption and tokenism; for example, there may be a sense that disruption slights colleagues and their work, highlighting differences that could make collaboration difficult. The second objective could reduce stigma by acknowledging the universality of lived experience and breaking down the ‘us versus them’ dichotomy that epitomizes stigma,^26^ producing an internal cultural shift. However, this could be criticized for avoiding a much‐needed critical appraisal of a system marred by inequities and tarnished by history. In the case of the third objective, it would be important that this not be the only focus and to monitor the initiative's authenticity to avoid unintended tokenism. Underrepresentation of certain lived experience groups^21^ could also introduce bias in all three approaches. Balance among objectives and engagement approaches should be considered when establishing the institutional objective.

## 4 HOW MIGHT LIVED EXPERIENCE ACADEMIC RESEARCHERS BALANCE THEIR LIVED EXPERIENCE AND RESEARCH IDENTITIES?

Identity is affected by many factors, such as personality, discipline, culture, ethnicity, self‐stigma, intersecting social determinants (i.e., gender, racism, socioeconomic status) and identification with lived experience. The career identity that individuals build for themselves contributes to their academic researcher role.^27^ Through our consultations, experience and examination of the literature, we have identified three dominant categories of lived experience academic researcher identities: (1) lived experience‐dominant identities in academic roles, where lived experience may be a key outward‐facing component of their identity and career trajectory; (2) academic‐dominant identities with accompanying lived experience that is applied to the work, where lived experience may be acknowledged and applied to their work, but where their career identities are built primarily as academics; (3) fully academic identities, with lived experience that is minimally acknowledged, where lived experience is present, but their identity is fully, or almost fully, defined as an academic researcher. Many may have mixed identities, emphasizing different aspects of their lived experience and academic identities in different social, cultural and professional contexts and at different career stages.^6^, ^13^ While identity is personal and cannot be prescribed, different identities have different implications for a lived experience academic initiative.

Academic researchers who identify primarily with lived experience may use this identity to generate change; they may be willing to fill an advocacy and spokesperson role, producing academic work that stimulates dialogue across disciplines and drives societal change. Open disclosure and public identification with lived experience within academia may foster a positive, supportive institutional atmosphere that encourages disclosure and reduces stigma, thereby encouraging appropriate help‐seeking when needed by members of academia.^20^, ^28^ However, academic researchers with lived experience‐dominant identities might not be fully accepted by the research community at large, despite—or because of—their unique scholarly contributions. In addition, bringing one's lived experience to the table on a daily basis is emotionally demanding^29^ and could lead to burnout.

Lived experience academic researchers who build their identities primarily as researchers may bring value by applying lived experience to their work, generating research that is relevant to individuals with lived experience. This role could be less personally demanding. However, such individuals may be criticized for not pursuing advocacy and their lived experience may be dismissed. Furthermore, a dominant researcher identity may be associated with hesitancy to disclose, as disclosure has long been discouraged.^30^ While the disclosure is a highly personal decision, without disclosure, there is no difference from the current situation.^31^

## 5 WHAT ROLES MIGHT BE HELD BY ACADEMIC RESEARCHERS WITH LIVED EXPERIENCE?

Various research roles highlighting lived experience may be created or acknowledged based on the institution's strategic priorities and the researchers' preferences. While academic researchers generally have substantial freedom to define their research agendas, they can also be appointed to specific roles within an institution. Two major stances we have identified on role definition are: (1) creating specific roles whereby the research position is defined by lived experience and the resulting research is expected to explicitly leverage lived experience; and (2) acknowledging lived experience among academic researchers, without necessarily expecting lived experience positionality to be represented in academic outputs. Regardless of the stance, various research roles and research platforms may be held. One important role would be the design and leadership of the lived experience initiative itself; from the standpoint of ‘nothing about us without us’,^32^ academic researchers with lived experience might be brought into the leadership of the lived experience academic initiative as early as possible in the research process. Lived experience academics may choose to establish platforms of research that focus on advocacy, critical appraisal of system inequities, other lived experience relevant issues and/or issues that are unrelated to lived experience but are informed by it; they may also bring their lived experience perspectives to projects across the institution. These roles do not have to be mutually exclusive.

Critically appraising a system as an advocate can be a powerful way of engendering system change by addressing service delivery, treatment and research processes. However, open criticism can be experienced as divisive rather than unifying and can thereby uphold rather than dismantle stigma; critical appraisal roles could also be tokenized if advocacy overshadows conventional research outputs and is not equally valued. In contrast, a research focus on lived experience‐relevant issues would produce outputs that advance the science of lived experience‐focused work.^20^ Bringing lived experience representation to projects across an institution can inform a range of research projects, increasing the relevance of the work generated across the institution; however, consistently bringing the same, critically important but often disputed issues to the table could lead to them being discounted. Using one's lived experience to inform research would help ensure that lived experience underpins the work on any number of topics. Openly acknowledging this dynamic among existing academic researchers might destigmatize lived experience. This could also go hand‐in‐hand with an initiative encouraging all academic researchers to be authentic allies, with training and support to create a safe culture of disclosure for academic researchers with lived experience.

## 6 HOW MANY LIVED EXPERIENCE ACADEMIC RESEARCHER(S) SHOULD BE ENGAGED?

An institution might choose to hire one or more individuals to fill researcher roles defined by lived experience. Alternatively, they may engage with lived experience more broadly, through a combination of lived experience research positions across career stages, lived experience opportunities for research trainees and junior research staff that may lead to more senior roles, lived experience committees, lived experience communities of practice and interinstitutional lived experience networks, in partnership with critical POR initiatives that engage lay people with lived experience.^33^ The decision will depend in part on the institution's strategic objective, its size, its commitment to lived experience research and the demand for this type of work within the institution and more broadly within academia and society.

Positioning a single person in this role would be the simplest option, at face value, but would come with a risk: this could be tokenizing, the individual would bear the burden alone, and a broad spectrum of voices would be missing. This approach may be seen as merely ‘checking a box’, without authentically addressing lived experience, stigma and EDI at the institutional level. This position might also be difficult or impossible to fill, given the issues raised above regarding disclosure, stigma and identity. In hiring for a position defined by lived experience, it would be important to carefully plan and consider how to ask about lived experience ethically and appropriately during an interview.

Bringing together multiple lived experience academic researchers in various roles would provide the opportunity for more meaningful change. This would diffuse the burden of lived experience representation across multiple academics and provide for mutual support—for example, through committees or communities of practice—as components of needed support mechanisms. The institution would benefit from a range of equity‐deserving voices representing different lived experience perspectives, incorporating various EDI factors. A broader approach might foster a sense of ‘belonging’ as the next step to EDI.^34^ It might also support sustainability, where lived experience is seen as ubiquitous and lived experience insights are viewed as a source of strength and inspiration. It could thereby affect broader institutional change, correcting a history of nonacceptance of lived experience within academia.^35^ This could be combined with researcher training and awareness raising to support academics with lived experience and build a culture of allies across disciplines. However, creating a multifaceted, multimember authentic lived experience academic initiative requires careful planning, capacity building, resources, readiness and substantial institutional commitment. This commitment must include explicit efforts to support the career development and the research and advocacy activities of the lived experience researchers.

## 7 WHERE WILL THE INSTITUTION FIND LIVED EXPERIENCE ACADEMIC RESEARCHERS?

Filling academic research positions defined by lived experience appears to be challenging, as our experience and consultations suggest that candidates who wish to hold such labelled positions are rare and context‐dependent. An institution may attempt to find lived experience academic researchers by creating regular external job postings or ‘headhunting’ such individuals.^17^ Alternatively, they may identify lived experience among the existing academic corpus. Another approach would be to build internal capacity by supporting established and emerging academic researchers with lived experience and embedding lived experience as an asset in all job postings across the institution.

External hires could bring new life and leadership into the institution. However, a posting framed as ‘lived experience researcher’ may attract individuals whose overall positionality does not align with the institution's objectives regarding the issues presented above. For example, a person whose goals are to revolutionize the academy may not find a sustainable home in the current academy. This type of posting could be stigmatized from the beginning. Furthermore, external hiring could be seen as overlooking members of the existing academic body with lived experience, discouraging disclosure and undermining the value that they bring.^29^

From a ‘grassroots’ perspective, academics with lived experience who are already woven throughout the organization may have the most practical insights into ways to leverage, amplify and support lived experience in that institution. However, it might not be possible to identify existing academics with the desired profiles, including a full range of EDI perspectives. This would be particularly limited if an institution is seeking someone who has not achieved academic success. Given the stigma, hesitancy to disclose,^27^, ^36^ fear of being ‘co‐opted’ by formal academic institutions and other barriers, any search method could prove challenging. A capacity‐building approach that values lived experience across the institution might gradually break down these barriers.^17^

## 8 CONCLUSIONS

Engaging with lived experience at more senior levels, that is, among academic researchers, holds the potential to enhance the movement towards POR approaches through truly lived experience research leadership.^1^, ^2^ However, given criticisms, controversies and conflicting opinions,^17^, ^20^ there are many factors to consider when planning to develop and implement a lived experience academic researcher initiative. Individuals and research institutions may uphold a wide range of positionalities with regard to the role and potential value of lived experience in research. The challenge, then, is to identify the approach that is most likely to be meaningful, productive and successful for the institution and the lived experience academic researchers themselves, given their respective goals, characteristics and broader social contexts.

Based on the sum of the reflections herein, we recommend a focus on developing well‐planned, diversified initiatives, backed by clear objectives and definitions, organizational commitment, researcher support mechanisms and clear risk mitigation strategies. Stigma considerations and support should be at the forefront, whether developing dedicated lived experience positions or supporting lived experience more broadly, or both.^22^ Importantly, we believe that these initiatives should be developed together with lived experience academic researchers themselves, leveraging POR learnings and past successes.^17^, ^18^

In developing a lived experience academic research initiative, institutional planners are encouraged to reflect first on their objectives and perspectives on lived experience, then on the ways lived experience might be identified, supported and valued within the organization. They should be transparent about their goals and values. Importantly, they should consistently and explicitly challenge the stigma that undermines lived experience within academia and in society at large. A well‐planned, diversified initiative backed by this type of in‐depth and ongoing reflection has the potential to stimulate a cultural shift within academia and beyond, breaking down the barriers of stigma, and guiding research activities towards meaningful issues with real‐world benefits. Ultimately, it can improve the quality of the evidence and the resulting healthcare, fostering a more inclusive academy and society.

## AUTHOR CONTRIBUTIONS

Lisa D. Hawke: *Conceived the manuscript, reviewed the literature and drafted the manuscript*. Natasha Y. Sheikhan: *Reviewed the literature, edited drafts of the manuscript and approved the final version*. Nev Jones, Mike Slade, Sophie Soklaridis, Samantha Wells and David Castle: *Edited drafts of the manuscript and approved the final version*.

## CONFLICT OF INTEREST

The authors have no conflict of interest to declare.

## Data Availability

Data sharing is not applicable to this article as no datasets were generated or analysed.
